# The population based cognitive testing in subjects with SARS-CoV-2 (POPCOV2) study: longitudinal investigation of remote cognitive and fatigue screening in PCR-positive cases and negative controls

**DOI:** 10.3389/fnhum.2024.1468204

**Published:** 2024-11-29

**Authors:** Alina von Etzdorf, Maja Harzen, Hannah Heinrichs, Henning Seifert, Stefan J. Groiß, Carolin Balloff, Torsten Feldt, Björn-Erik Ole Jensen, Tom Lüdde, Michael Bernhard, Alfons Schnitzler, Klaus Goebels, Jörg Kraus, Sven G. Meuth, Saskia Elben, Philipp Albrecht

**Affiliations:** ^1^Department of Neurology, Medical Faculty and University Hospital Düsseldorf, Heinrich Heine University Düsseldorf, Düsseldorf, Germany; ^2^Department of Neurology, Maria-Hilf-Clinics Mönchengladbach, Mönchengladbach, Germany; ^3^Department of Gastroenterology, Hepatology and Infectious Diseases, Medical Faculty and University Hospital Düsseldorf, Heinrich Heine University Düsseldorf, Düsseldorf, Germany; ^4^Emergency Department, Faculty of Medicine, Heinrich Heine University, Düsseldorf, Germany; ^5^Institute for Clinical Neuroscience and Medical Psychology, Faculty of Medicine, Heinrich Heine University Düsseldorf, Düsseldorf, Germany; ^6^Düsseldorf Health Department, Düsseldorf, Germany; ^7^Department of Laboratory Medicine, Paracelsus Medical University and Salzburger Landeskliniken, Salzburg, Austria

**Keywords:** cognitive testing in COVID-19, fatigue screening COVID-19, depression screening COVID-19, COVID-19, cognitive impairment, remote cognitive testing, depression, post covid

## Abstract

**Background:**

The majority of people infected with severe acute respiratory syndrome coronavirus 2 (SARS-CoV-2) only show mild respiratory symptoms. However, some patients with SARS-CoV-2 display neurological symptoms. Data on the exact prevalence and course of cognitive symptoms are often limited to patient reported outcomes or studies recruited at specialized centers.

**Methods:**

For this prospective, non-interventional population based POPCOV2 study, 156 subjects who performed SARS-CoV-2 testing in the Düsseldorf metropolitan area at public test centers between December 2020 and February 2022 were recruited by handouts. SARS-CoV-2-positive and negatively tested subjects were included within the first seven days after the PCR test results. Cognitive testing was performed at baseline during home quarantine and after 4–6 as well as 12–14 weeks of follow-up. Individuals were examined remotely by videocalls using the Symbol Digit Modalities Test (SDMT) and the Montreal Cognitive Assessment (MoCA) in addition to the Brief Fatigue Inventory (BFI) and the Beck Depression Inventory-Fast Screen (BDI-FS).

**Results:**

At baseline, the SARS-CoV-2-positive group presented with higher levels of fatigue in the BFI. In both the SARS-CoV-2-positive and SARS-CoV-2-negative groups, some subjects presented attention and memory deficits, defined as a z-score < −1,65 on the SDMT or < 26 points on the MoCA (SDMT: 22.9% in the positive and 8.8% in the negative group, *p* = 0.024; MoCA: 35.6% in the positive and 27.3% in the negative group, *p* = 0.313). MoCA and SDMT improved over time in both groups. For MoCA scores, a significant difference between the two groups was only seen at the first follow-up. SDMT z-scores did not differ at any time between the groups.

**Conclusion:**

These results support previous evidence that mild SARS-CoV-2 infections are associated with increased fatigue. However, we found relevant rates of cognitive impairment not only in the infected but also in the control group. This underlines the importance of including a control group in such investigations.

## Introduction

Since its first appearance in 2019, SARS-CoV-2 (severe acute respiratory syndrome coronavirus 2) has ravaged humanity in the worldwide COVID-19 pandemic but the severity of COVID-19 has meanwhile decreased and the virus has become endemic. While COVID-19 primarily affects the respiratory system it can also lead to cognitive symptoms including confusion, difficulty concentrating, and memory problems, often referred to as “brain fog” (Hampshire et al., 2024). Symptoms can persist even after recovery, impacting daily functioning (Ceban et al., 2022; Premraj et al., 2022). Post-COVID-19 conditions are characterized by structural and functional impairments of several organs. Not only pulmonary and cardiological damage, but also neurological and psychiatric deficits can be recognized (Quan et al., 2023). Neurocognitive deficits such as concentration, memory and executive function have been reported and were most frequently observed in hospitalized patients. An analysis of the electronic health records of more than 236,000 people infected with COVID-19 also showed that non-hospitalized patients were significantly more likely to develop psychiatric disorders such as mood swings, anxiety, insomnia and psychosis. Furthermore, it is essential to note that these effects are not limited to people with severe acute symptoms or those with a long history of COVID-19, but can also occur in milder cases without persistent symptoms (Zhao et al., 2023).

A recent two-year retrospective cohort study of over one million participants infected with COVID-19 found that the risk of cognitive deficits after six months was higher than in the control group, with a hazard ratio of 1.36 (1.33–1.39) (Quan et al., 2023). This risk remained elevated at the end of the two-year follow-up period of the disease. Another longitudinal cohort study in China with 3,233 COVID-19 patients reported that severe COVID-19 disease was associated with a higher risk of early-onset cognitive decline (six months after discharge), late-onset cognitive decline (12 months after discharge) and progressive cognitive decline than in control subjects (Hampshire et al., 2024). A study from England investigated whether participants with persistent symptoms (≥12 weeks) after the onset of infection had objectively measurable global cognitive deficits. Study participants who had persistent symptoms resolved after COVID-19 had objectively measured cognitive function comparable to participants with shorter duration of symptoms, although a short COVID-19 duration was still associated with small post-recovery cognitive deficits (Hampshire et al., 2024).

Several previous studies have performed cognitive testing to investigate minor and/or major neurocognitive disorders after Covid-19 using short screening measures such as the Montreal Cognitive Assessment (MoCA) or the Mini Mental Status Examination (MMSE). One of these studies reported a higher rate of decline on the MoCA in individuals who were seropositive for COVID-19 compared to those who were seronegative (Del Brutto et al., 2021). Another study examined the performance on MoCA and the Frontal Assessment Battery (FAB) in hospitalized patients diagnosed with severe COVID-19 in the post-critical acute phase of the disease (Beaud et al., 2021). Alemanno et al. (2021) found cognitive impairment in 80% of hospitalized patients infected with COVID-19 using MoCA and MMSE, while Ortelli et al. (2021) found significantly lower MoCA scores in patients with COVID-19 compared to healthy controls (Ortelli et al., 2021).

Woo et al. (2020) used a modified telephone interview to determine that patients recovering from COVID-19 performed significantly worse than healthy controls, particularly on measures of short-term memory, attention, concentration, and language (Woo et al., 2020). Overall, the available data suggest that COVID-19 infection may be associated with cognitive dysfunction even months after the acute illness.

However, SARS-CoV-2 is not the only virus associated with cognitive impairment and “brain fog”; EBV, HSV and HTLV are other examples (Burrell et al., 2017). Symptoms may occur during and after the viral infection, but can also recur over the course of up to several months. Patients with symptoms persisting more than four weeks after the acute phase of a SARS-CoV-2 infection classify as “long COVID”^1^. We recently examined the diagnostic and prognostic value of electrophysiological and cognitive assessments in hospitalized COVID-19 patients without previous neurological disease and identified relevant rates of peripheral and central nervous system impairment as well as cognitive deficits (Balloff et al., 2023; Costa et al., 2023).

We report the results of the prospective, population based POPCOV2 study, which aimed to investigate the prevalence and dynamics of cognitive impairment during and after the acute infection in population-based cohorts of SARS-CoV-2 positive and negative people from a representative urban area in Germany. Including people with negative SARS-CoV-2 test results as a control cohort allowed us to assess a possible recruitment bias, resulting from complaint-dependent willingness to participate in the study.

## Methods

### Cohorts and recruitment

The inclusion criteria were > =18 years of age and a positive or negative result of a polymerase chain reaction (PCR) test for SARS-CoV-2 within seven days from enrollment and baseline examination. Our test subjects all have German nationality and were recruited by information leaflets handed out by testing centers in Düsseldorf, Germany. We acknowledge the fact that it would have been of interest to investigate a possible influence of the vaccination status of our participants. However, the vaccination status was, unfortunately, not assessed in the POPCOV2 study.” The recruitment period was December 2020 to February 2022.

### Remote cognitive testing

Study participants were examined in their home setting up to four times by means of end-to-end encrypted videoconference via the online video portal Click-Doc (AG CMD, 2021). The baseline assessment (initial testing) was conducted within the first week of a SARS-CoV-2 PCR test. The first follow-up 4–6 weeks after baseline testing, the second follow-up after 12–14 weeks from baseline, and the fourth follow-up was performed 6 months after baseline. Study participants were tested using the Symbol Digit Modalities Test (SDMT) and the Montreal Cognitive Assessment (MoCA). The Symbol Digit Modalities Test is a screening instrument for the presence of neurocognitive deficits. In this test nine different symbols are linked to the numbers 1 to 9. In a timed examination, subjects are then asked to write the correct number under the corresponding symbol (Smith, 1982). The Montreal Cognitive Assessment is a rapid screening instrument for mild cognitive impairment (MCI) or dementia. It is a 1-page, 30-item test which takes about 10 min to complete. It includes tasks on short-term memory recall, visual–spatial abilities, executive functions, phonemic fluency, verbal abstraction, attention, concentration and working memory. Language and orientation to time and place are also assessed (Nasreddine et al., 2005). SDMT was used to investigate information processing speed and MoCA was used as a global screening for cognitive impairment (Smith, 1982). Z-scores of < −1 were considered as suggestive of an impaired information processing speed, and z-scores < − 1.65 as evidence for clinically relevant cognitive dysfunction. A score of <26 out of 30 was considered as suggestive of mild cognitive impairment (Nasreddine et al., 2005). Both test procedures have been validated for video-based administration (Jennings et al., 2021; Smith, 1982). The test sheets were presented to the subjects on the screen and responses were recorded by the investigators on the test record forms. For the visuospatial tests subjects drew test objects on a paper sheet and presented it to the examiner on camera for assessment. Furthermore, additional questionnaires were included to examine the subjects’ mood by using the Beck Depression Inventory Fast Screen (BDI-FS) and the Brief Fatigue Inventory (BFI) to asses fatigue (Fossati et al., 2011; Beck et al., 2000). Individual symptoms of the test subjects were recorded using unstructured oral interviews.

### Statistical testing

Descriptive statistics were performed using R version 4.3.2 and SPSS version 28, *p*-values <0.05 were considered significant. To test normal distribution in the population samples, we applied the Shapiro–Wilk test and Kolmogorov–Smirnov test. Group comparisons were performed using the Mann–Whitney U test due to ordinal scaling or non-normally distributed parameters. Comparisons of categorical parameters were performed using Fisher’s exact test. We adjusted the *p*-values calculated from the Mann–Whitney U and Fisher’s exact tests using the Benjamini-Hochberg procedure.

To verify the results obtained with the raw MoCA values, we transformed the individual MoCA values of the SARS-CoV-2 positive and negative subjects into age-and education-related z-scores as previously described. To account for the relevant factors of potential influence and the longitudinal nature of the data we have performed an additional mixed effects linear model (MLM) analysis using SARS-CoV-2 status, age, sex, education status and time interaction as fixed and patient ID as random effects. *p*-values are provided for the different tests as indicated and values below 0.05 were considered significant. In addition, we performed Spearman correlation analyses between both neurocognitive tests (MoCA, zSDMT). We also correlated the test questionnaires on depressive symptoms and fatigue (BDI-FS, BFI) with each other and with the neurocognitive tests. In addition, we z-transformed the rho values of the four measurement points. We then used a two-sample *z*-test to examine whether the pairwise differences of the z-transformed rho values deviate significantly from the null hypothesis.

## Results

### Demographics of probands at baseline examination

At baseline from December 2020 till February 2022, we observed differences between the symptoms of SARS-CoV-2 positive and negative subjects. Thus, typical cold symptoms such as cough, rhinorrhea, cephalgia, and body ache were more frequently reported by the SARS-CoV-2 positive subjects in contrast to the negatively tested control group. Hyposmia and ageusia were also strikingly more pronounced in the SARS-CoV-2 infected subjects compared to the control group. In addition, the SARS-CoV-2 positive subjects rated their subjective cognitive performance and their subjective feeling of illness during the acute phase of infection as more severe than the SARS-CoV-2 negative subjects (see Table 1).

**Table 1:** Demographics of probands at baseline examination.

	SARS-CoV-2 positive	SARS-CoV-2 negative	*p*-value
N	167	69	
Male, *N* (%)	95 (56.9%)	27 (39.1%)	0.024
Female, *N* (%)	72 (43.1%)	42 (60.9%)	
Diverse, *N* (%)	0 (0%)	0 (0%)	
Age, mean	39.6	40.7	0.827
Education subgroups^a^			
Level 1, *N* (%)	1 (0.6%)	1 (1.5%)	0.501
Level 2, *N* (%)	16 (9.8%)	5 (7.6%)	
Level 3, *N* (%)	1 (0.6%)	2 (3.0%)	
Level 4, *N* (%)	17 (10.4%)	10 (15.2%)	
Level 5, *N* (%)	128 (78.5%)	48 (72.7%)	
Cough, *N* (%)	90 (53.9%)	11 (15.9%)	< 0.0001
Rhinorrhoea, *N* (%)	81 (48.8%)	8 (11.6%)	< 0.0001
Sore Throat, *N* (%)	30 (18.0%)	11 (16.2%)	0.851
Fever, *N* (%)	15 (9.0%)	1 (1.4%)	0.057
Cephalgia and body ache, *N* (%)	69 (41.6%)	8 (11.6%)	< 0.0001
Hyposmia and Ageusia, *N* (%)	64 (38.2%)	3 (4.3%)	< 0.0001
Subjective cognitive performance^b^, mean	2.446	1.771	0.002
Subjective feeling of sickness^b^, mean	2.504	1.714	< 0.0001

a Education divided into subgroups: 1 – lower secondary school leaving certificate. 2 – intermediate school leaving certificate. 3 – higher education entrance qualification (A-levels). 4 – higher education entrance qualification (A-levels) plus apprenticeship. 5 – higher education entrance qualification (A-levels) plus any kind of university education.

b Scale of 1–6 (equivalent to German school grades with 1 being the best and 6 being the worst possible outcome).

### Results of the SDMT examination

**Figure 1:**
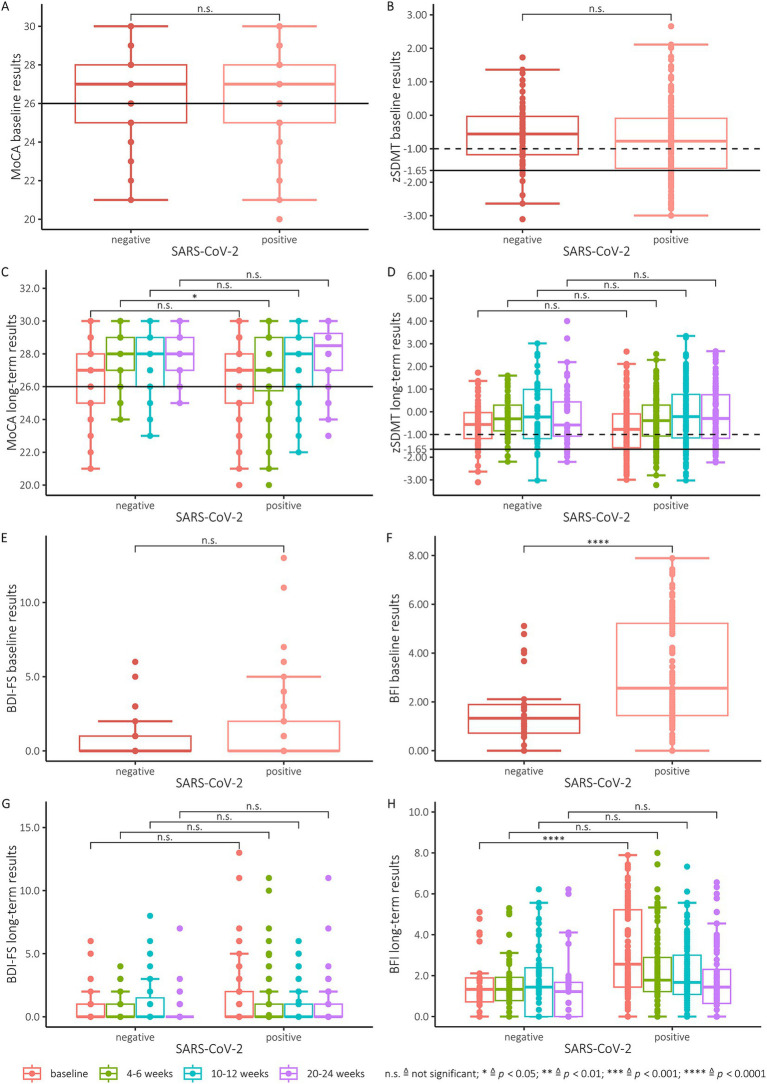
**(A)** MoCA baseline results. All scores below the black line (26 and below) indicate cognitive impairment. **(B)** Baseline results of the SDMT (converted to zSDMT). The dotted line illustrates the cut-off at −1 and the solid line the cut-off at −1.65. **(C)** MoCA results of all participants tested positive for SARS-CoV-2 and the negative control group for all 4 testing points. All scores below the line (26 and below) indicate cognitive impairment. **(D)** zSDMT-score of the participants tested positive for SARS-CoV-2 and the control group for all 4 testing points. The dotted line illustrates the cut-off at −1 and the solid line the cut-off at −1.65. **(E)** Baseline results of SARS-CoV-2 positive participants and the negative control group for the BDI-FS. For interpretation the manual suggests scores 10–21 are indicative of severe depression, moderate (7–9), mild (4–6) and minimal depression (0–3). **(F)** Baseline results of SARS-CoV-2 positive participants and the negative control group for the BFI. Scores are categorized as mild (1–3), moderate (4–6), and severe (7–10). A global fatigue score can be found by average score obtained on each test item. **(G)** Performance of SARS-CoV-2 positive participants and the negative control group for baseline testing and all follow-ups for the BDI-FS. **(H)** Performance of SARS-CoV-2 positive participants and the negative control group for baseline testing and all follow-ups for the BFI.

The Shapiro Wilk and Kolmogorov Smirnov tests for normal distribution showed that the SDMT was normally distributed. SARS-CoV-2 positive and negative subjects had an average zSDMT score of −0.75 (SD = 1.14) and − 0.56 (SD = 0.92) respectively at baseline and showed an improvement to a z-score of −0.38 (SD = 1.07) for positive and − 0.2 (SD = 0.92) for negative subjects at the second test, with the improvement in positive subjects proving significant (*p* = 0.14, *p* < 0.05 respectively) (Figure 1B). Thus, the means of both groups remained above the cut-off score for a clinically relevant deficit of z < −1.65. The groups with positive and negative PCR testing did not differ at baseline (*p* = 0.315). On the individual level, 38 (22.8%) positive and 6 (8.8%) negative subjects fell below this cut-off score at baseline (Figure 1B) and 21 (13.8%) positive and 3 (4.8%) negative subjects at the second assessment (Figure 1D). During the course of the study, an overall improvement in SDMT performance between baseline testing and testing after 6 months could be observed (Figure 1D). SARS-CoV-2 positive subjects showed an improvement to a z-score of −0.75 to −0.15 after 6 months (Figure 1D). Negative controls improved to a score of −0.56 to −0.17 after 6 months (Figure 1D). At 6-month follow-up, 9 (9%) positive and 5 (10.9%) negative subjects were observed with test performances below the cut-off (Figure 1D). When comparing the results of SARS-CoV-2 positive and negative subjects, no statistically significant differences of z-scores could be found both at baseline (*p* = 0.35), after second (*p* = 0.45) or third (*p* = 0.94) testing and after 6 months (*p* = 0.94) regarding the cognitive processing speed as measured by the SDMT (Figure 1D). The mixed effects linear model analysis revealed a significant effect of time since baseline (*p* < 0.001) on the zSDMT values while SARS-CoV-2 status, age, sex and education level did not (*p* = 0.223, *p* = 0.392, *p* = 0.376 and *p* = 0.409, respectively)

### Results of the MoCA examination

The test for normal distribution showed that the MoCA test was not normally distributed.

SARS-CoV-2 positive and negative subjects had an average MoCA score of 26.3 (SD = 2.2) and 26.6 (SD = 2.1), respectively, at baseline (Figure 1A) and also showed significant improvements to a score of 26.9 (SD = 2.3) for positive and 27.8 (SD = 1.7) for negative subjects at the second examination (*p* < 0.05, *p* = <0.01 respectively) (Figure 1C). However, there was no difference of MoCA-scores between the two groups at baseline (*p* = 0.35). In contrast, at the first follow-up after four weeks the SARS-CoV-2 positive group performed worse. At this test time, a significant difference was found between the two groups of test subjects (*p* = 0,035) (Figure 1C). During the course of the study, an overall improvement in MoCA performance between baseline and testing at 6 months was observed (Figure 1C). At baseline testing, 36.5% of SARS-CoV-2 positive subjects had a MoCA below 26 points, with 12% remaining under this cut-off after 6 months (fourth testing). However, in the control group, 26% had a MoCA score < 26 points at baseline testing, and 2% improved their MoCA above the cut-off score after 6 months. The SARS-CoV-2-negative subjects also improved from a mean of 26.6 (baseline) to a mean of 28.2 at 6 months (fourth test). We observed no significant difference in cognitive impairment between SARS-CoV-2 subjects and the control group at both the 3-month and 6-month time points. In addition to these analyses of the MoCA raw values we additionally transformed these to age-and education-based z-scores. No difference was found between the SARS-CoV-2 positive and negative subjects when comparing the MoCA Z-scores at baseline and after 3 and 6 months. Similar to the results obtained with the raw MoCA scores, a significant difference (*p* < 0.001) (Figure 1C) was found between the two groups at the second examination after 4 weeks. The mixed effects linear model analysis revealed a significant effect of time since baseline (*p* = 0.001) on the MoCA values while SARS-CoV-2 status, age, sex and education level did not (*p* = 0.146, *p* = 0.077, *p* = 0.053 and *p* = 0.264, respectively).

### Results of the BFI and BDI-FS examination

The test for normal distribution showed that both the BFI and the BDI-FS are not normally distributed.

We observed a significant difference between SARS-CoV-2 positive and negative subjects in BFI test scores at baseline (*p* < 0.0001) (Figure 1F). In the first follow-up after 4–6 weeks (*p* = 0.08), the second follow-up after 3 month (*p* = 0.69) and the final follow-up after 6 month (*p* = 0.28) no significant differences between the two groups were detected (Figure 1H). The SARS-CoV-2 positive subjects revealed higher mean scores at baseline with a mean of 3.2 (SD = 1.3), decreasing to a mean of 1.8 (SD = 1.6) at the last follow-up after 6 months. In comparison, the control group achieved a mean value of 1.5 (SD = 1.3) at baseline, which was consecutively reduced to a mean of 1.3 (SD = 1.5) after 6 months. For the BDI-FS, the SARS-CoV2 positive and negative groups did not differ (Figures 1E,G) Similarly, the SARS-CoV-2-positive subjects achieved higher mean scores at baseline, decreasing from a mean of 1.3 (SD = 2.1) to a mean of 0.6 (SD = 1.5) at the last follow-up at 6 months. The mean values of the negative control group were similar, but still moderately lower, with a mean of 0.8 (SD = 1.4) at baseline examination and a mean of 0.4 (SD = 1.2) at the last follow-up after 6 months.

The mixed effects linear model analysis revealed no significant effects of SARS-CoV-2 status, age, time, sex and education level since baseline on the BDI-FS values (*p* = 0.347, *p* = 0.745, *p* = 0.400, *p* = 0.805 and *p* = 0.198 respectively). However, the mixed effects linear model analysis revealed significant effects of SARS-CoV-2 status (*p* < 0.05) and time (*p* = 0.035) since baseline on the BFI values while age, sex and education level did not (*p* = 0.638, *p* = 0.561 and *p* = 0.301 respectively).

### Correlation analyses

**Figure 2:**
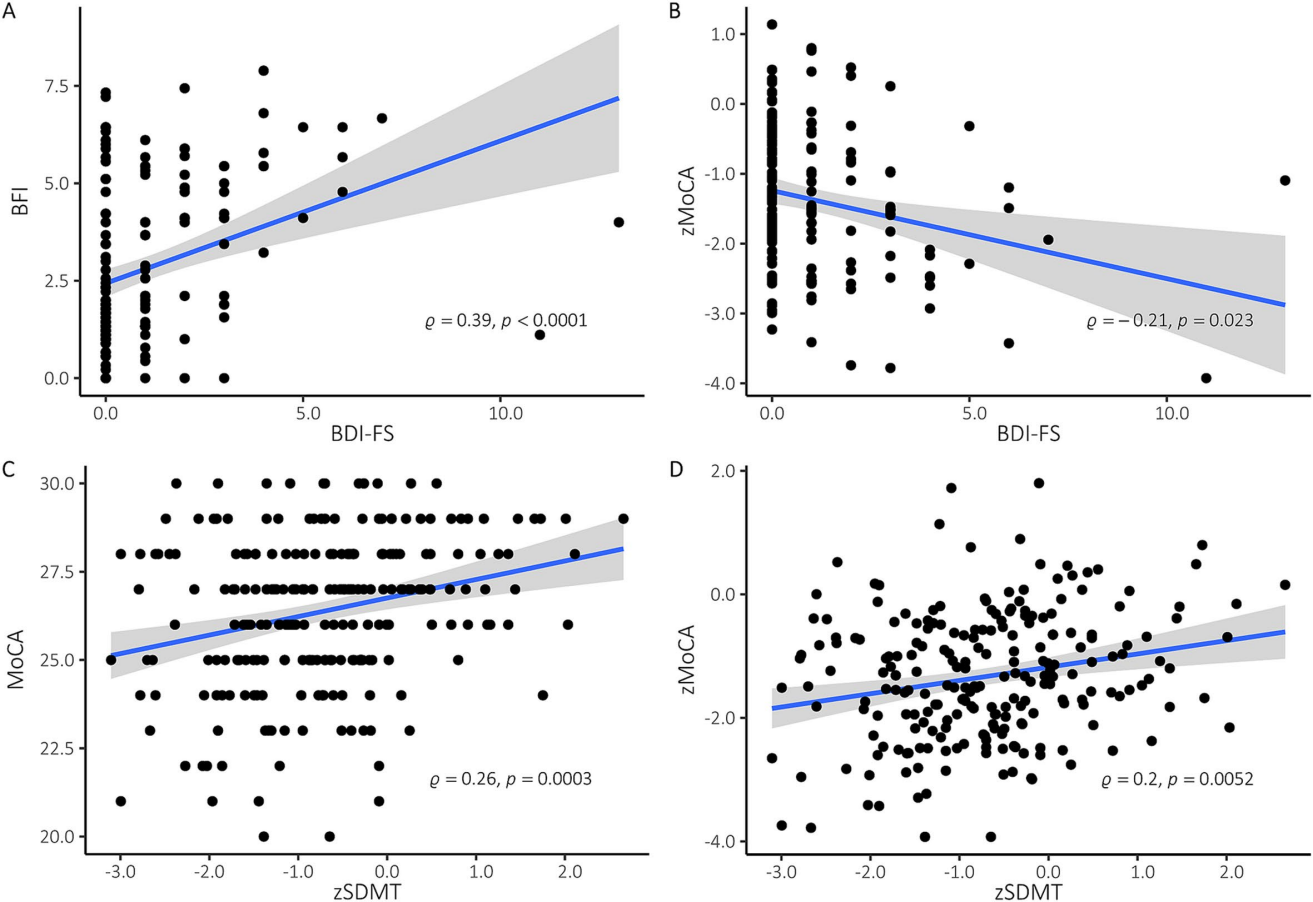
**(A)** Spearman correlation at baseline examination of BDI-FS and BFI. Linear regression with 95% confidence intervals, spearman rho and *p* values are provided. Higher scores in BDI-FS und BFI indicate more severe depression and fatigue, respectively. **(B)** Spearman correlation at baseline examination of zMoCA and BDI-FS. Linear regression with 95% confidence intervals, spearman rho and *p* values are provided. *Z*-values of < −1 are considered an indication of impaired information processing speed, *z*-values < − 1.65 as an indication of clinically relevant cognitive dysfunction. **(C)** Spearman correlation at baseline examination of MoCA and zSDMT. Linear regression with 95% confidence intervals, spearman rho and *p* values are provided. All MoCA-scores below 25 indicate cognitive impairment. **(D)** Spearman correlation at baseline between zMoCA and zSDMT. Linear regression with 95% confidence intervals, spearman rho and *p* values are provided.

The Spearman correlation between the zSDMT and MoCA scores showed a significant correlation at baseline (*ρ* = 0.26, *p* < 0.0001), as well as at the second (*ρ* = 0.32, *p* < 0.0001), third (*ρ* = 0.26, *p* < 0.001) and fourth (*ρ* = 0.2, *p* < 0.01) follow-up tests (Figure 2C). We performed separate Spearman correlation analyses to investigate the associations of zSDMT with zMoCA at the different timepoints: They correlated significantly at baseline (*ρ* = 0.2, *p* < 0.01) (Figure 2D), first (ρ = 0.26, *p* < 0.001) and second (ρ = 0.17, *p* < 0.05) follow-up. However, both groups did not correlate in the third follow-up examination after six months (ρ = 0.09, *p* = 0.42). A Z-test comparing the correlation coefficients for the different timepoints did not reveal significant differences between the timepoints of the baseline and the subsequent follow-up examinations.

Furthermore, BDI-FS and BFI correlated significantly with each other at all four time points of assessment (Figure 2A).

Z-test analyses revealed no difference in correlation coefficients between baseline (ρ = 0.39, *p* < 0.0001), second (ρ = 0.34, *p* < 0.0001) third (ρ = 0.34, *p* < 0.0001) and fourth (ρ = 0.46, *p* < 0.0001) examination. In addition, only the baseline examination showed a significant correlation between BDI-FS and zMoCA (ρ = −0.21, *p* < 0.01) and between BFI and zSDMT (ρ = −0.18, *p* < 0.05) (Figure 2B).

## Discussion

In contrast to several previous reports, this study focuses on non-hospitalized SARS-CoV-2-positive persons. One of the main advantages of the POPCOV2 study’s design was the remote testing, which the test subjects could easily carry out from home, which largely facilitated testing during the acute infectious phase under quarantine conditions. Moreover, patients were closely followed-up over a period of six months and were compared to a SARS-CoV-2 negative control group. This allowed us to investigate several important albeit up to now often unaddressed aspects. On the one side, we compared the cognitive performance of subjects with recent positive SARS-CoV-2 PCR tests to negatively tested persons. On the other side, the long-term development of fatigue and depressive symptoms were prospectively investigated and compared to negative controls. Out of a total of 246 test subjects, 146 subjects (61.9%) completed all four tests in their entirety. The third test was achieved by 188 test subjects (79.6%). By using validated neuropsychological screening procedures our study extends upon previous reports focusing on subjective patient reported outcomes (Barilaite et al., 2024).

Short cognitive screening tools commonly used to examine minor and major neurocognitive impairments after COVID-19. Comprehensive cognitive examinations of COVID-19 survivors are significantly more rare. A recent study presented the results of a complete neuropsychological examination of patients with severe COVID-19 symptoms who were treated in the intensive care unit and then tested by telephone (Whiteside et al., 2021). The examination showed impairments in memory encoding, verbal fluency, and the onset of new psychiatric symptoms such as depression and anxiety. Zhou et al. (2020) found that reaction times and alertness had slowed in people who had recovered from COVID-19 in China, in a short, iPad-based test battery focused on attention and processing speed. This could be related to inflammatory processes (Zhou et al., 2020). In addition, a large online study (*N* = 81,337) reported cognitive deficits in individuals who reported a confirmed or suspected COVID-19 infection, even if they no longer had symptoms and after adjusting for many confounding factors (Hampshire et al., 2021). These results suggest persistent cognitive changes in COVID-19 survivors. However, despite these findings, data on the cognitive profile of individuals in the chronic phases of recovery are warranted. Such data would be helpful to better understand risk factors for cognitive impairment after COVID-19 infection, as well as the cognitive areas most commonly affected.

A large study from Bangladesh with 2,198 participants investigated the prevalence of long COVID symptoms and possible associated risk factors. In this cohort, the prevalence of long COVID symptoms 31 weeks after diagnosis was 16.1%. Female gender, rural residence, previous functional impairment and smoking were identified as risk factors for persistence (Hossain et al., 2021). Likewise in Bangladesh, persistent COVID-19 symptoms associated with depression were investigated in 1002 people using an online questionnaire. Forty-eight percent of participants were classified as moderately to severely depressed. A multivariate regression analysis revealed that depression during COVID-19 was positively associated with lower family income, poor health and sleep disturbances (Islam et al., 2021). In Wuhan, China, a study investigated the 1-year course of cognitive changes in older COVID-19 survivors. A total of 1,438 COVID-19 survivors and 438 control subjects were included in the final follow-up. Cognitive changes during the first and second 6-month follow-up periods were assessed using the Informant Questionnaire on Cognitive Decline in the Elderly and the Telephone Interview on Cognitive Status, respectively. In this cohort study, COVID-19 survival was associated with an increased risk of long-term cognitive decline (Liu et al., 2022).

Furthermore, including a PCR-negative control group allowed us to investigate the bias resulting from a voluntary population-based recruitment. Very similar to the results of the recently published cognitive results of the REACT study (Hampshire et al., 2024) we observed similar mild cognitive impairment in the SARS-CoV-2 positive subjects as in the negative controls. We found cognitive deficits in the MOCA in 20.4% of the positive subjects at baseline. The rate of patients with cognitive deficits then continuously decreased after 4 to 5 weeks (19.9%), to 12.3% after 10 to 12 weeks and finally only 5.4% after 6 months in SARS-CoV-2 positive subjects.

However, in the control group, the rate of subjects with cognitive deficits also continuously declined. It has to be mentioned, that the control group in our study started at a lower level of cognitive impairment at baseline, which did, however, not differ significantly from the SARS-CoV-2 positive subjects for most timepoints. This assumption is supported by the fact that the differences between the two groups did reach significance at the second follow up. We acknowledge that our study may have been underpowered to detect these subtle differences. We assume that our control group with SARS-CoV-2 negative subjects contains a substantial number of patients with infections other than SARS-CoV-2, which may also be associated with cognitive deficits. This is in line with our observation that also the SARS-CoV-2 negative control group ameliorated over time. Our findings, therefore, suggest that other respiratory infections also lead to a cognitive impairment. A narrative literature review shows the effects of COVID-19, but also of six other acute viral infections (HSV-1, VZV, JEV, WNV, Influenza A and B), on human cognition. Various viral pathogens have been shown to impair human cognition through different pathogenetic mechanisms. Some of these pathogens can cause long-term cognitive impairment, including parenchymal brain damage due to direct CNS infection or indirect mechanisms leading to impaired brain function, such as hypercoagulable states and neuroinflammation (Damiano et al., 2022).

The rather high rate of cognitive impairment in the SARS-CoV-2 negative control group may be an indicator for a relevant recruitment bias, since people with subjective cognitive impairment may have been more likely to feel attracted to participation in the study. In consequence of the restrictions and limitations during the COVID-19 pandemic our study has some limitations: The test setting only allowed for a brief cognitive assessment using screening tests. A personalized comprehensive cognitive examination was not feasible. Furthermore, we were not able to control for typical sources of bias: We could not assure that all participants were examined at the same time of the day and we could not control the setting and environment of cognitive testing (e.g., distraction resulting from the environment during remote home testing). These sources of bias were, however, identical for positive and negative subjects. It is also important to point out that, despite the use of alternating questionnaires, the improvement of the test subjects during the course of the measurements could be attributed to a learning effect, e.g., by adapting the strategy for completing the task (Vaniprabha et al., 2022). Therefore, symptom improvement is not necessarily the only factor responsible for a better test result at follow up. In order to minimize potential learning effects we have used alternative versions of the SDMT and MOCA. However, in line with previous studies (Méndez et al., 2021; Panagea et al., 2024; Vyas et al., 2022) the performance of the both positive and negative group ameliorated over time, which may be suggestive of learning effects linked to retesting. These could be explained, e.g., by optimizing the strategy to solve the SDMT and MOCA tasks. An alternative and/or additional explanation for the cognitive improvement observed in our study could be the fact that both the SARS-CoV-2-positive and the negatively tested subjects reported flu-like symptoms during the first assessment. It is more than reasonable to assume that the cognitive performance increased with the decrease of such symptoms. One of the main advantages of the POPCOV2 study’s design was the remote testing, which the test subjects could easily carry out from home, which largely facilitated testing during the acute infectious phase under quarantine conditions.

In consequence of the restrictions and limitations during the COVID-19 pandemic our study has some limitations: The test setting only allowed for a brief cognitive assessment using screening tests. A personalized comprehensive cognitive examination was not feasible. We were not able to control for typical sources of bias: We could not assure that all participants were examined at the same time of the day and we could not control the setting and environment of cognitive testing (e.g., distraction resulting from the environment during remote home testing). The educational level of the participants of our study was higher than that of the general population in Germany and thus not representative regarding this aspect. However, these sources of bias were identical for the SARS-CoV-2 positive and negative subjects. Furthermore, we report not only the raw values but also z-scores of our cognitive test data (see Supplementary data for zMOCA), which adjust the values to the performance of healthy controls with a similar level of education, age and sex. Even if our test subjects have a higher level of education, some of them were cognitively impaired in comparison to healthy controls of similar education, age and sex.

Another limitation of our study is that we only asked the subjects about their mother tongue and nationality, but not about their ethnic background.

Overall, the present study did not reveal evidence of cognitive impairment in the SARS-CoV-2 positive subjects with mild respiratory symptoms compared to a matched control group from the same population. At the same time, our study did reveal significant differences regarding fatigue and depression in SARS-CoV-2 positive subjects during the acute phase of infection. Thus, it is reasonable to assume, that the fatigue reported by SARS-CoV-2 positive patients is not simply related to exhaustion in the context of cognitive impairment but may, instead, be linked to or associated with depressive symptoms. At the same time, the depressive symptoms and fatigue might have influenced the rather high rates of MoCA and SDMT scores below the cut-off at baseline. This would also explain the improvement of these scores in the long-term analysis since we also observed an improvement in the results of BDI-FS and BFI.

Our results support previous evidence that mild SARS-CoV-2 infections are associated with increased fatigue (Rao et al., 2021; Hartung et al., 2022). Our finding of relevant rates of cognitive impairment also in the negative control group demonstrates the importance of including a control group in such investigations, which is in line with previous reports (Quan et al., 2023). In conclusion, our results support previous evidence that mild SARS-CoV-2 infections are associated with increased fatigue. The finding of our cognitive testing demonstrating relevant rates of cognitive impairment also in the negative control group demonstrates the importance of including a control group in such investigations.

## Data Availability

The raw data supporting the conclusions of this article will be made available by the authors, without undue reservation.

## Footnotes

^1^ https://www.cdc.gov/coronavirus
