# Supplementary material for: The population based cognitive testing in subjects with SARS-CoV-2 (POPCOV2) study: longitudinal investigation of remote cognitive and fatigue screening in PCR-positive cases and negative controls
Source: Front Hum Neurosci. 2024 Nov 29;18:1468204. doi: 10.3389/fnhum.2024.1468204 (PMC11638161; doi:10.3389/fnhum.2024.1468204)
Supplement: Supplementary file 1 [file Data_Sheet_1.docx]

Supplementary Material

Figure 1


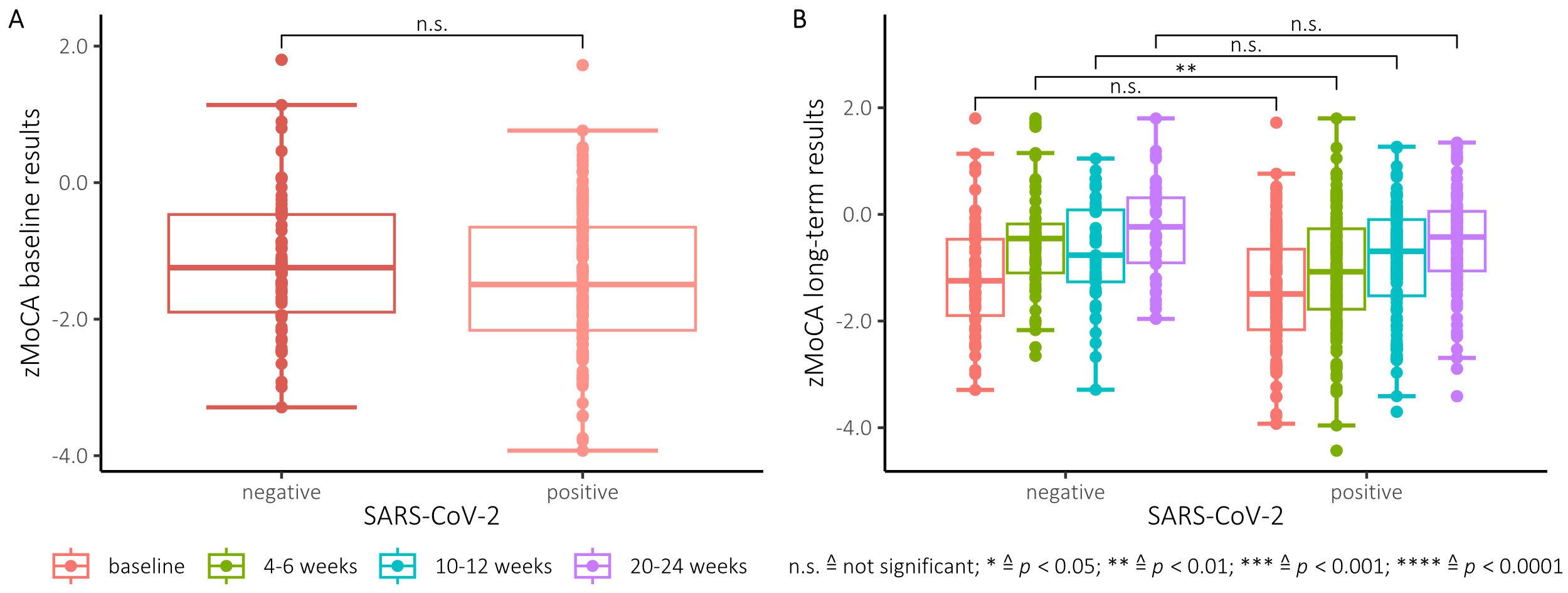


**Figure 1A**: zMoCA baseline results.

**Figure 1B**: zMoCA results of all participants tested positive for SARS-CoV-2 and the negative control group for all 4 testing points.

Figure 2


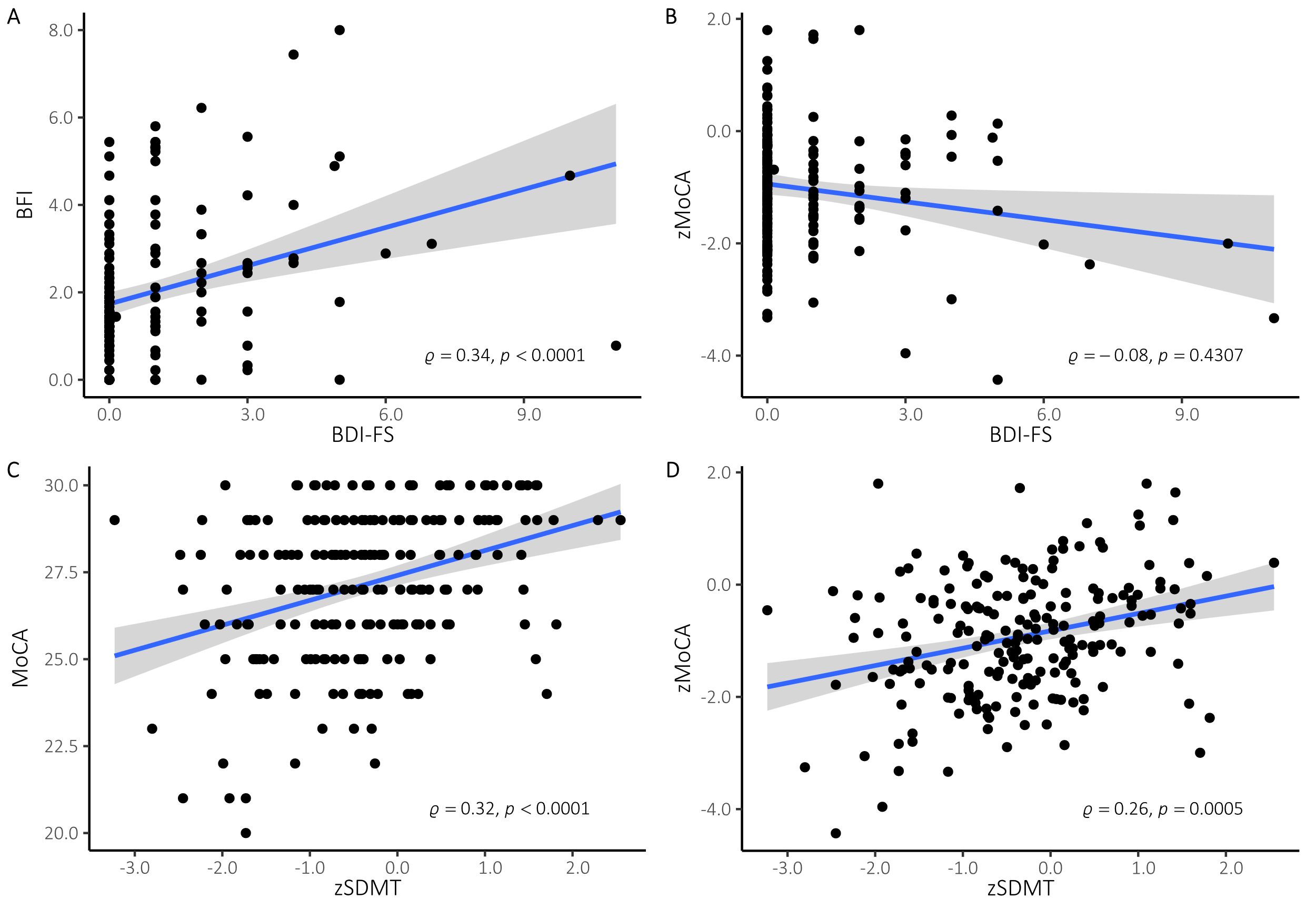


**Figure 2A:** Spearman correlation at second testing of BDI-FS and BFI. Linear regression with 95% confidence intervals, spearman rho and p values are provided. Higher scores in BDI-FS und BFI indicate more severe depression and fatigue, respectively.

**Figure 2B:** Spearman correlation at second testing examination of zMoCA and BDI-FS. Linear regression with 95% confidence intervals, spearman rho and p values are provided. Z-values of < -1 are considered an indication of impaired information processing speed, z-values < - 1.65 as an indication of clinically relevant cognitive dysfunction.

**Figure 2C:** Spearman correlation at second testing of MoCA and zSDMT. Linear regression with 95% confidence intervals, spearman rho and p values are provided. All MoCA-scores below 25 indicate cognitive impairment.

**Figure 2D:** Spearman correlation at second testing between zMoCA and zSDMT. Linear regression with 95% confidence intervals, spearman rho and p values are provided.

Figure 3


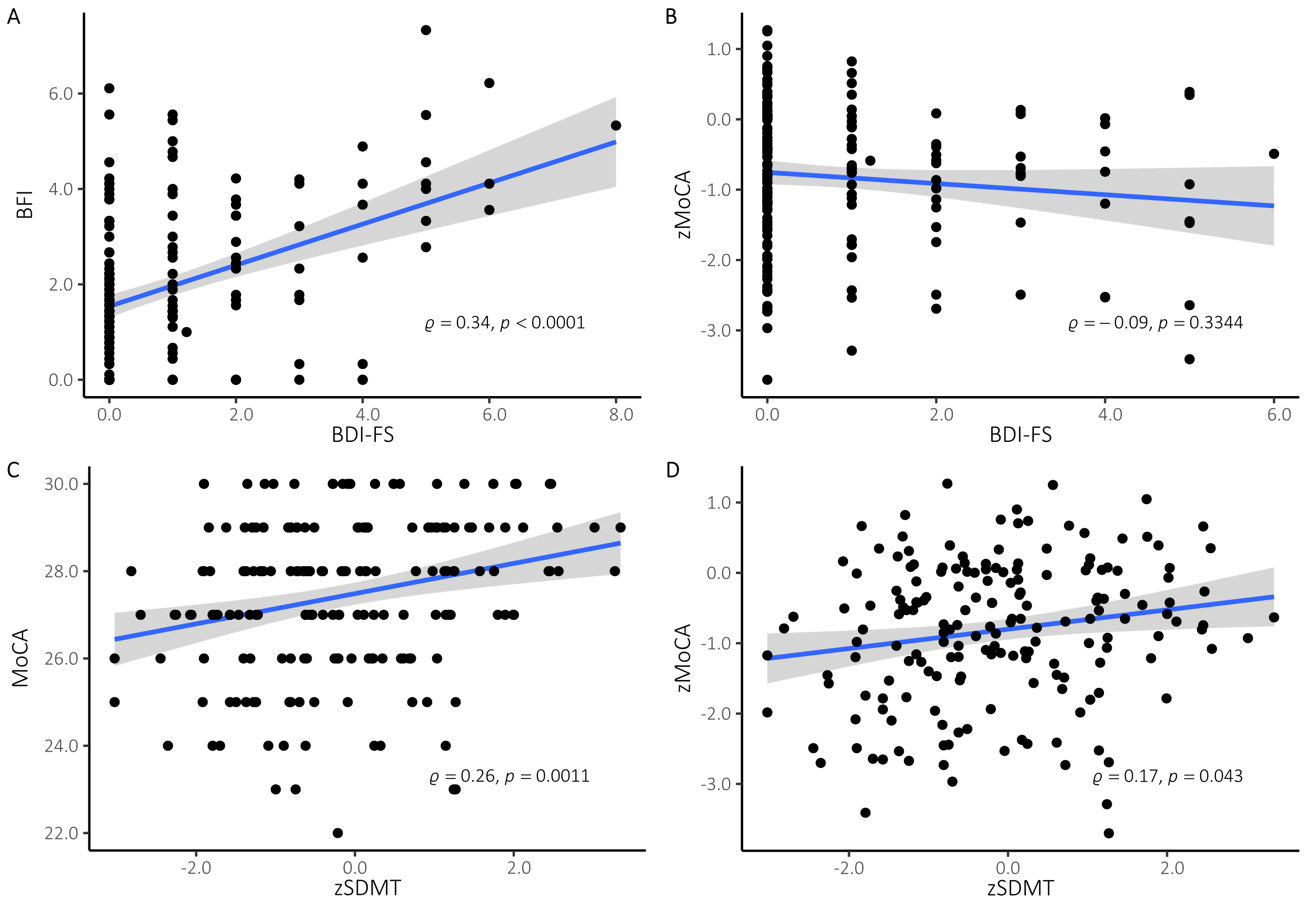


**Figure 3A:** Spearman correlation at third testing of BDI-FS and BFI. Linear regression with 95% confidence intervals, spearman rho and p values are provided. Higher scores in BDI-FS und BFI indicate more severe depression and fatigue, respectively.

**Figure 3B:** Spearman correlation at third testing examination of zMoCA and BDI-FS. Linear regression with 95% confidence intervals, spearman rho and p values are provided. Z-values of < -1 are considered an indication of impaired information processing speed, z-values < - 1.65 as an indication of clinically relevant cognitive dysfunction.

**Figure 3C:** Spearman correlation at third testing of MoCA and zSDMT. Linear regression with 95% confidence intervals, spearman rho and p values are provided. All MoCA-scores below 25 indicate cognitive impairment.

**Figure 3D:** Spearman correlation at third testing between zMoCA and zSDMT. Linear regression with 95% confidence intervals, spearman rho and p values are provided.

Figure 4


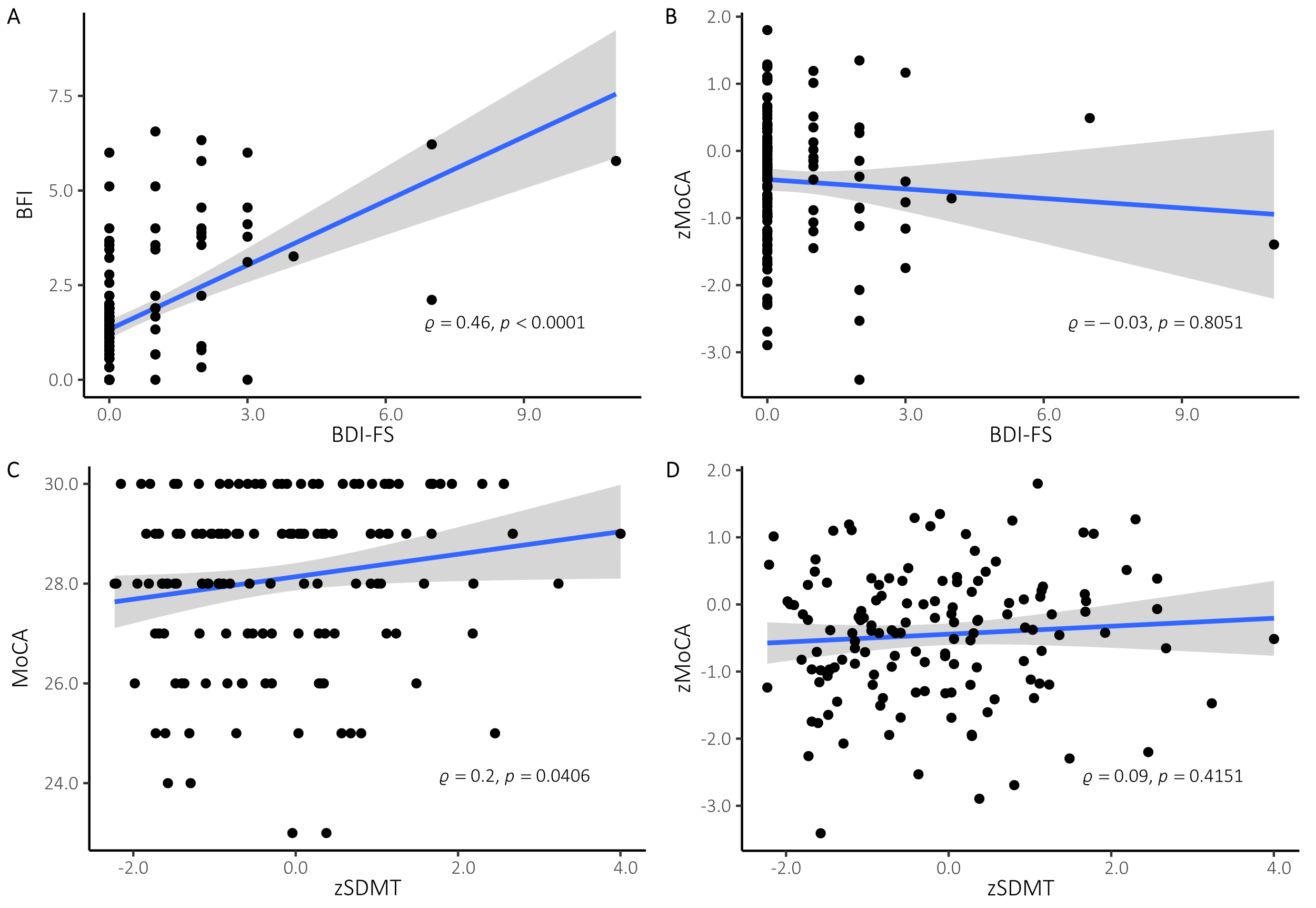


**Figure 4A:** Spearman correlation at fourth testing of BDI-FS and BFI. Linear regression with 95% confidence intervals, spearman rho and p values are provided. Higher scores in BDI-FS und BFI indicate more severe depression and fatigue, respectively.

**Figure 4B:** Spearman correlation at fourth testing examination of zMoCA and BDI-FS. Linear regression with 95% confidence intervals, spearman rho and p values are provided. Z-values of < -1 are considered an indication of impaired information processing speed, z-values < - 1.65 as an indication of clinically relevant cognitive dysfunction.

**Figure 4C:** Spearman correlation at fourth testing of MoCA and zSDMT. Linear regression with 95% confidence intervals, spearman rho and p values are provided. All MoCA-scores below 25 indicate cognitive impairment.

**Figure 4D:** Spearman correlation at fourth testing between zMoCA and zSDMT. Linear regression with 95% confidence intervals, spearman rho and p values are provided.

Figure 5


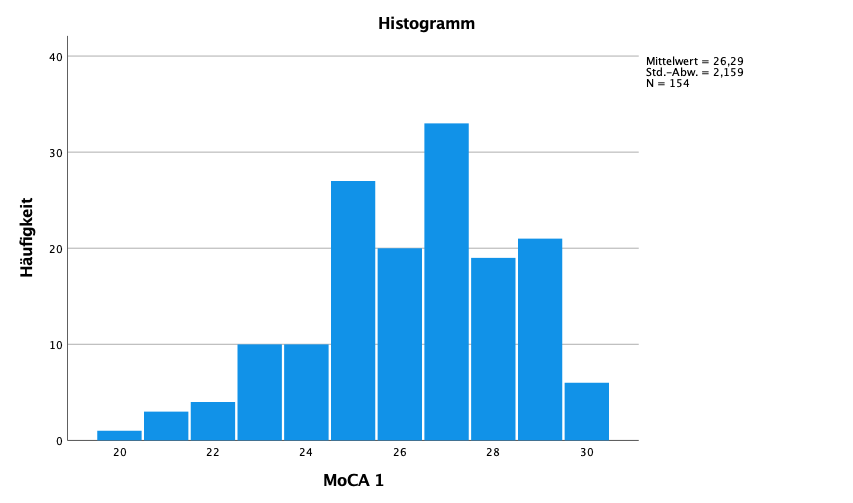

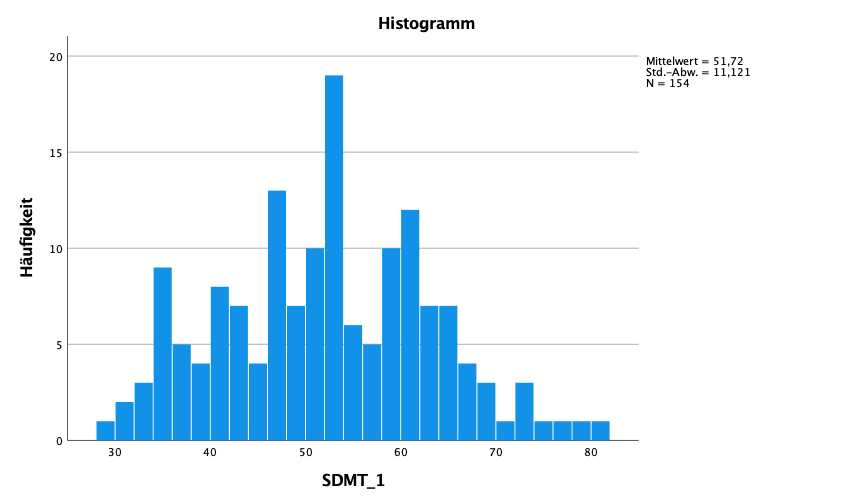

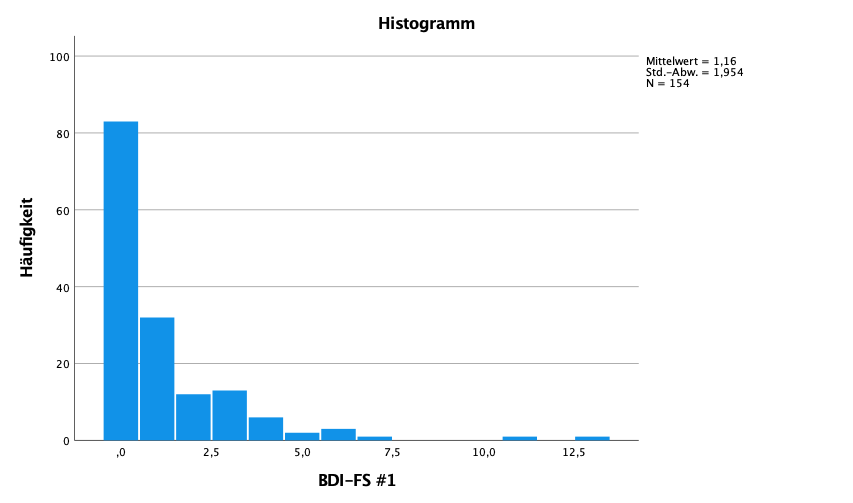

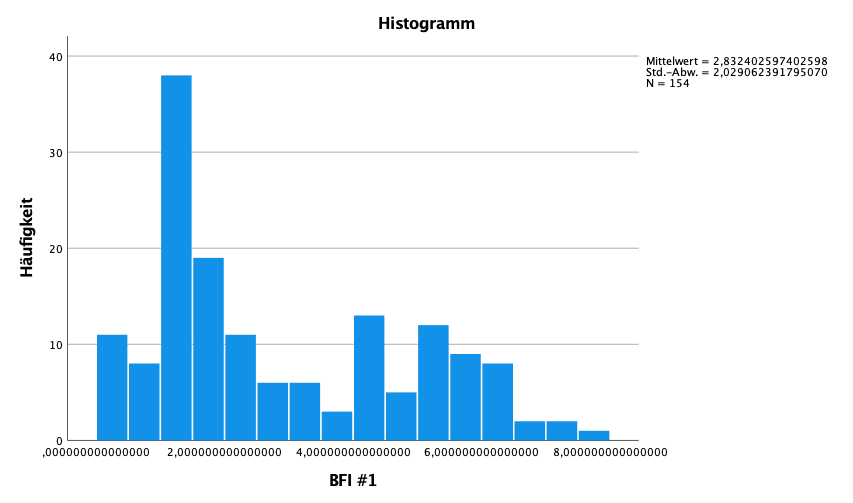


**Figure 5:** Histograms showing the distribution of the test procedures MocA, SDMT, BFI and BDI-FS

# Metrics of Distribution

| Variable | Median | Q1 | Q3 | IQR |
| --- | --- | --- | --- | --- |
| Age | 39 | 29 | 50 | 21 |
| BDI-FS (baseline) | 0 | 0 | 2 | 2 |
| BDI-FS (visit 2) | 0 | 0 | 1 | 1 |
| BDI-FS (visit 3) | 0 | 0 | 1 | 1 |
| BDI-FS (visit 4) | 0 | 0 | 0 | 0 |
| BFI (baseline) | 2 | 1.33 | 4.78 | 3.45 |
| BFI (visit 2) | 1.56 | 1.11 | 2.67 | 1.56 |
| BFI (visit 3) | 1.67 | 0.89 | 2.78 | 1.89 |
| BFI (visit 4) | 1.385 | 0.0825 | 2 | 1.9175 |
| Education | 5 | 5 | 5 | 0 |
| Education (SDMT) | 2 | 2 | 2 | 0 |
| Education years | 15 | 15 | 15 | 0 |
| MoCA (baseline) | 27 | 25 | 28 | 3 |
| MoCA (visit 2) | 28 | 26 | 29 | 3 |
| MoCA (visit 3) | 28 | 26 | 29 | 3 |
| MoCA (visit 4) | 28 | 27 | 29 | 2 |
| SDMT (baseline) | 51.5 | 43 | 59 | 16 |
| SDMT (visit 2) | 54 | 48 | 62 | 14 |
| SDMT (visit 3) | 54 | 47 | 65.5 | 18.5 |
| SDMT (visit 4) | 54 | 47 | 65 | 18 |
| Subjective Cognitive Performance (baseline) | 2 | 2 | 3 | 1 |
| Subjective Cognitive Performance (visit 2) | 2 | 1 | 3 | 2 |
| Subjective Cognitive Performance (visit 3) | 2 | 1 | 3 | 2 |
| Subjective Cognitive Performance (visit 4) | 2 | 1 | 2 | 1 |
| Subjective Feeling of Sickness (baseline) | 2 | 2 | 3 | 1 |
| Subjective Feeling of Sickness (visit 2) | 2 | 1 | 2 | 1 |
| Subjective Feeling of Sickness (visit 3) | 2 | 1 | 2 | 1 |
| Subjective Feeling of Sickness (visit 4) | 1 | 1 | 2 | 1 |
| zMoCA (baseline) | -1.33154 | -2.08723 | -0.62357 | 1.463661 |
| zMoCA (visit 2) | -0.91268 | -1.61533 | -0.20767 | 1.407662 |
| zMoCA (visit 3) | -0.69726 | -1.45346 | 0.007846 | 1.46131 |
| zMoCA (visit 4) | -0.38352 | -1.04626 | 0.153844 | 1.200106 |
| zSDMT (baseline) | -0.69964 | -1.45964 | -0.05946 | 1.40018 |
| zSDMT (visit 2) | -0.3501 | -0.94637 | 0.30737 | 1.25374 |
| zSDMT (visit 3) | -0.21638 | -1.15286 | 0.94329 | 2.09615 |
| zSDMT (visit 4) | -0.294 | -1.15286 | 0.67812 | 1.83098 |

# Statistics on CoViD/Non-CoViD groups

| Variable | Visit | CoViD | | *n*_1_ | *n*_2_ | *p* | Significance |
| --- | --- | --- | --- | --- | --- | --- | --- |
| BDI-FS | Baseline | negative | positive | 35 | 121 | 0.351 | n.s. |
| BDI-FS | Visit 2 | negative | positive | 44 | 117 | 0.465 | n.s. |
| BDI-FS | Visit 3 | negative | positive | 51 | 137 | 0.692 | n.s. |
| BDI-FS | Visit 4 | negative | positive | 46 | 100 | 0.676 | n.s. |
| BFI | Baseline | negative | positive | 35 | 121 | 1.72∙10^-5^ | **** |
| BFI | Visit 2 | negative | positive | 44 | 117 | 0.0812 | n.s. |
| BFI | Visit 3 | negative | positive | 51 | 136 | 0.692 | n.s. |
| BFI | Visit 4 | negative | positive | 46 | 100 | 0.2844 | n.s. |
| MoCA | Baseline | negative | positive | 69 | 167 | 0.351 | n.s. |
| MoCA | Visit 2 | negative | positive | 65 | 152 | 0.0357 | * |
| MoCA | Visit 3 | negative | positive | 51 | 137 | 0.953 | n.s. |
| MoCA | Visit 4 | negative | positive | 46 | 100 | 0.935 | n.s. |
| SDMT | Baseline | negative | positive | 68 | 166 | 0.351 | n.s. |
| SDMT | Visit 2 | negative | positive | 65 | 152 | 0.4488 | n.s. |
| SDMT | Visit 3 | negative | positive | 51 | 136 | 0.7185 | n.s. |
| SDMT | Visit 4 | negative | positive | 46 | 99 | 0.6975 | n.s. |
| zMoCA | Baseline | negative | positive | 66 | 163 | 0.351 | n.s. |
| zMoCA | Visit 2 | negative | positive | 62 | 148 | 0.00531 | ** |
| zMoCA | Visit 3 | negative | positive | 49 | 133 | 0.692 | n.s. |
| zMoCA | Visit 4 | negative | positive | 43 | 98 | 0.676 | n.s. |
| zSDMT | Baseline | negative | positive | 68 | 166 | 0.351 | n.s. |
| zSDMT | Visit 2 | negative | positive | 63 | 152 | 0.4488 | n.s. |
| zSDMT | Visit 3 | negative | positive | 51 | 136 | 0.953 | n.s. |
| zSDMT | Visit 4 | negative | positive | 46 | 99 | 0.935 | n.s. |
